# Case Report: Primary sciatic leiomyosarcoma in a patient with neurofibromatosis type 1

**DOI:** 10.3389/fonc.2026.1682808

**Published:** 2026-05-07

**Authors:** Longgang Chen, Meiling Xu, Kai Cheng, Lingya Cui, Xuefei Mou, Changhui Li

**Affiliations:** 1 Department of Sports Medicine, Rizhao People’s Hospital, Rizhao, Shandong, China; 2 Oncology Department, Rizhao People’s Hospital, Rizhao, Shandong, China; 3 Operation Department, Rizhao People’s Hospital, Rizhao, Shandong, China; 4 Rehabilitation Department, Rizhao People’s Hospital, Rizhao, Shandong, China

**Keywords:** leiomyosarcoma, neurofibromatosis type 1, sciatic nerve, smooth muscle cells, surgery

## Abstract

This case report discusses a rare occurrence of primary sciatic leiomyosarcoma in a patient with type 1 neurofibromatosis. The patient had a gradually enlarging lump in her right thigh, which was diagnosed as a leiomyosarcoma after surgery. The association between neurofibromatosis type 1 (NF1) and leiomyosarcoma has not been well-established. However, smooth muscle tumors in NF1 patients are often multitudinous, and careful follow-up is crucial due to the potential development of leiomyosarcomas. The case report highlights the diagnostic and management challenges presented by this rare occurrence. The patient underwent surgical resection of the tumor(14×11cm), including a portion of the proximal and distal sciatic nerve, achieving an R0 margin,followed by radiation therapy (60 Gy in 30 fractions). The postoperative period was uneventful, with no recurrence or metastasis observed during the six-month follow-up period.

## Introduction

Leiomyosarcoma (LMS) is a rare malignancy that accounts for less than 1% of all adult cancers. It is a subtype of soft tissue sarcoma (STS) which encompasses a heterogeneous group of tumors with over 175 molecular subtypes. LMS is one of the more common subtypes of STS, comprising up to 10-20% of all sarcomas (1). The median age at diagnosis is around 50–60 years, and the tumor has a slight male predominance (2). Leiomyosarcomas originate from smooth muscle cells and mainly occur in abdominal organs with smooth muscle components, including the uterus, stomach, small intestine, and retroperitoneum. They can also arise in other locations that contain smooth muscle, such as skin and skeletal muscles. As smooth muscle components are found in vascular tissues, leiomyosarcoma can potentially develop in any location that contains vascular tissues (3). However, leiomyosarcoma of the sciatic nerve, as seen in this case, is extremely rare, with only a few cases reported in the literature (4, 5).

Neurofibromatosis type 1 (NF1) is an autosomal dominant genetic disorder that affects approximately 1 in 3,500 individuals worldwide (6). It is characterized by the development of multiple benign tumors of the nervous system, as well as other clinical manifestations such as café-au-lait spots, skin fold freckling, and Lisch nodules. Individuals with NF1 also have an increased risk of developing malignant tumors, particularly malignant peripheral nerve sheath tumors (MPNSTs) (7). To date, there has only been a few reported cases of leiomyosarcoma in NF1 patients (8–11).

In this case report, we present the clinical, radiological, and pathological features of a 56-year-old female patient with NF1 who presented with a primary sciatic leiomyosarcoma. We also discuss the diagnostic and therapeutic challenges associated with this rare tumor in the context of NF1. This case highlights the importance of considering leiomyosarcoma in the differential diagnosis of tumors along the course of the sciatic nerve in patients with NF1, and emphasizes the need for multidisciplinary management in the treatment of such cases.

## Case description

**Figure 1:**
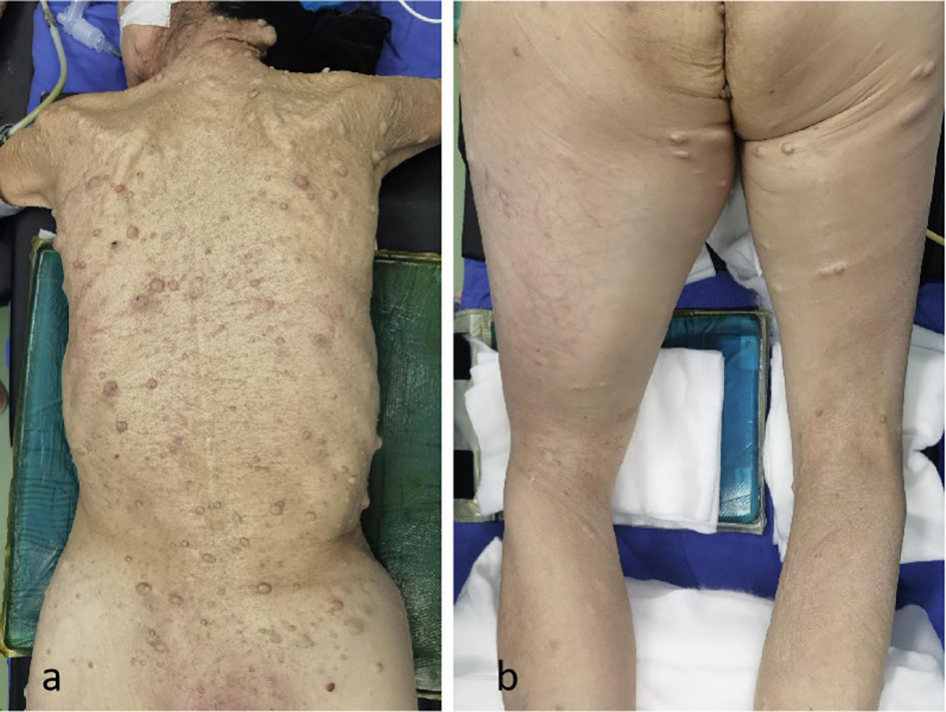
Multiple neurofibromas in the trunk area **(a)**, significant swelling on the inner and posterior aspects of the thigh with a palpable hard mass underneath the skin **(b)**.

**Figure 2:**
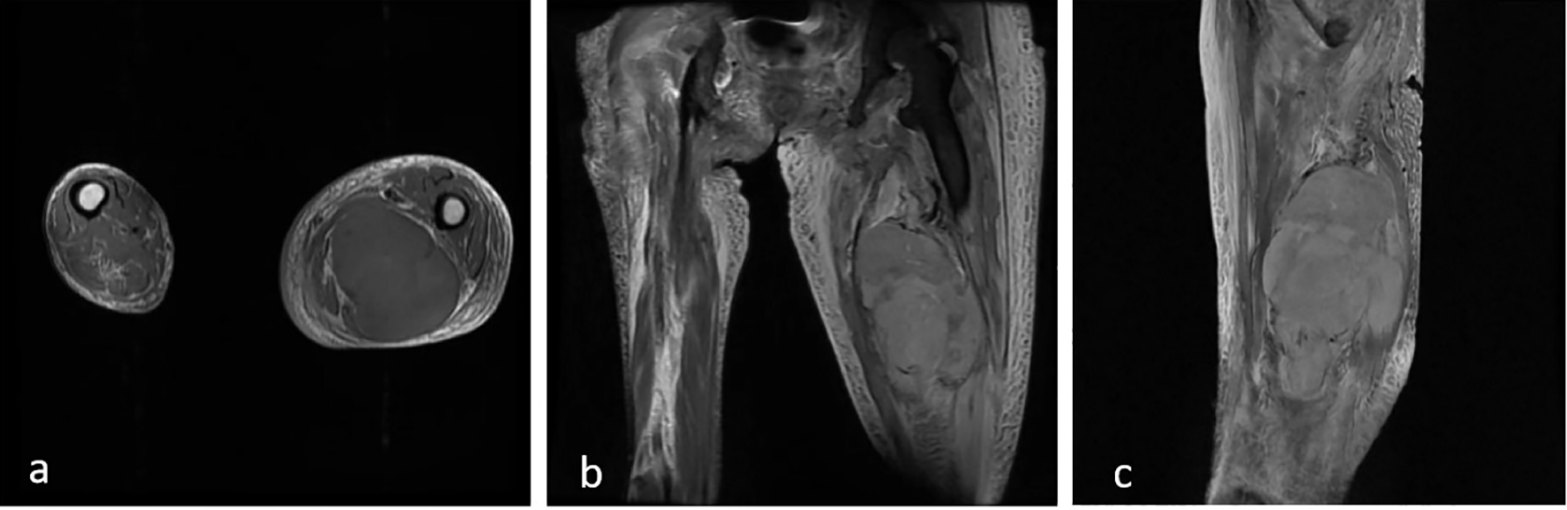
Sequential MRI in axial **(a)**, coronal **(b)** and sagittal **(c)** views showed a huge mass in the posteromedial aspect of the thigh.

A 56-year-old woman with a history of type-1 neurofibromatosis and a family history of the condition presented with a gradually enlarging lump in her right thigh (**Figure 1a**). The mass had been slowly growing over the past 8 months, with a noticeable acceleration in size during the 2 months prior to presentation. Physical examination showed significant swelling on the inner and posterior aspects of the thigh with a palpable hard mass underneath the skin (**Figure 1b**). The patient had sciatic nerve dysfunction in her right lower limb, including an inability to move the right ankle (BMRC grade 0) and sensory loss, but could flex and extend the right knee joint (BMRC grade 4). She reported constant, dull pain in the right thigh and posterior aspect of the right leg with a VAS score of 6/10, which was described as neuropathic in nature. Tinel’s sign was positive along the course of the sciatic nerve. The area below the right knee joint was significantly swollen due to tumor compression, which obstructed blood and lymphatic flow, resulting in deep vein thrombosis. Ultrasound and MRI examinations showed a tumor along the sciatic nerve in the thigh (**Figure 2**). MRI revealed a heterogeneous mass with irregular margins, areas of necrosis, and heterogeneous enhancement, which raised suspicion for malignancy rather than a benign neurofibroma.

**Figure 3:**
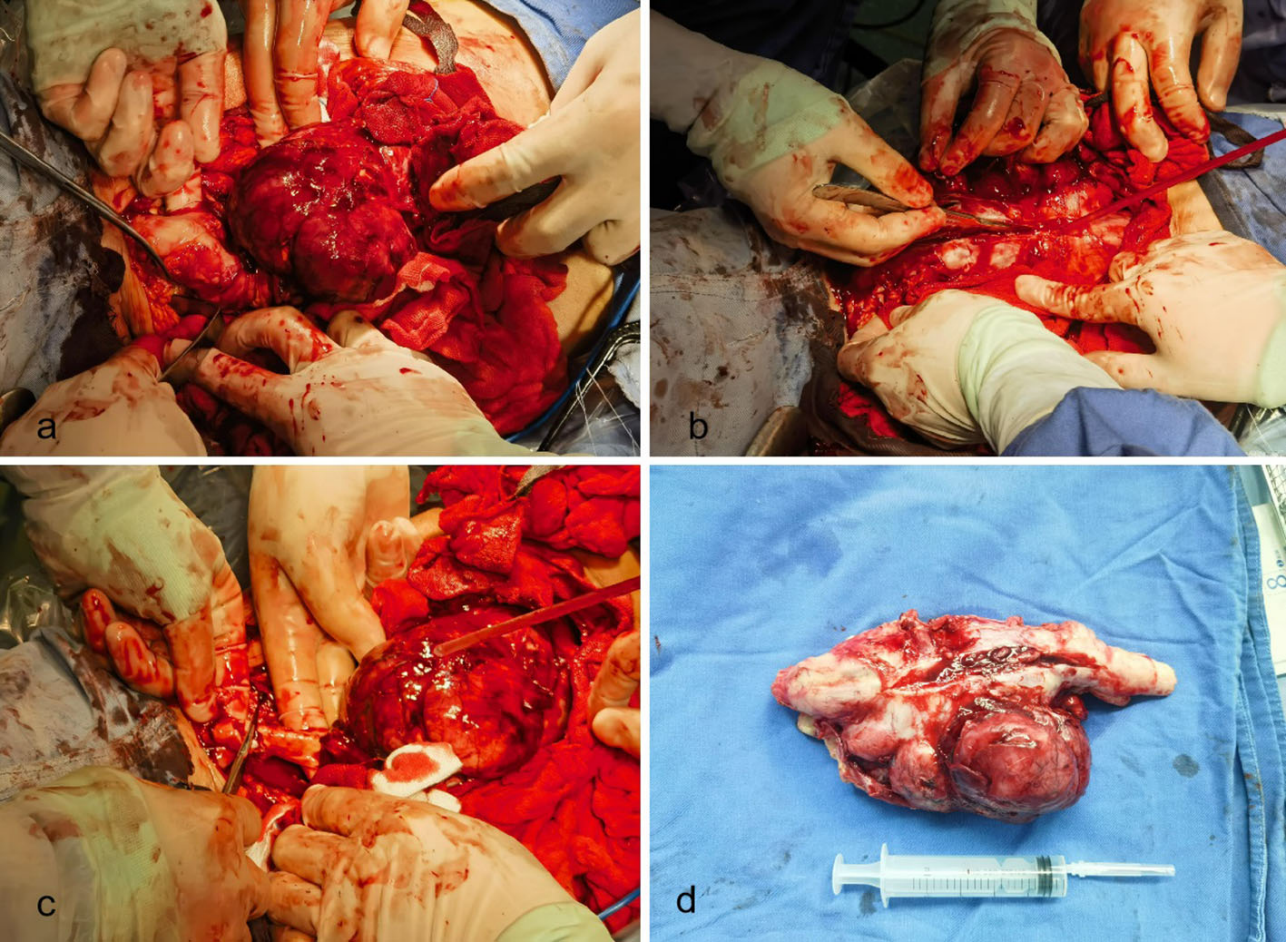
**(a)** A 14×11cm tumor with lobulated growth infiltrating along the sciatic nerve. **(b)** Attempts to peel the tumor from the nerve were unsuccessful. **(c)** The tumor is resected together with the sciatic nerve **(d)** Excised tumor and sciatic nerve.

Before surgery, the patient had anemia and hypoalbuminemia, which were difficult to correct with blood transfusion and albumin supplementation. During surgery, a 14×11cm tumor with lobulated growth infiltrating along the sciatic nerve was found (**Figure 3a**). The tumor was firmly adhered to the nerve tissue with no discernible dissection plane. Intraoperative neurophysiological monitoring was not available at our institution. Attempts to peel the tumor from the nerve were unsuccessful (**Figure 3b**), and given the extensive involvement and the malignant nature of the tumor confirmed by intraoperative frozen section, the decision was made to perform an en bloc resection of the tumor along with the involved segment of the sciatic nerve. The upper resection margin was at the level of the ischial tuberosity, and the lower margin was at the bifurcation of the sciatic nerve into the tibial and common peroneal nerves (**Figures 3c, d**). The patient lost approximately 400 mL of blood during the procedure and did not require a blood transfusion.

**Figure 4:**
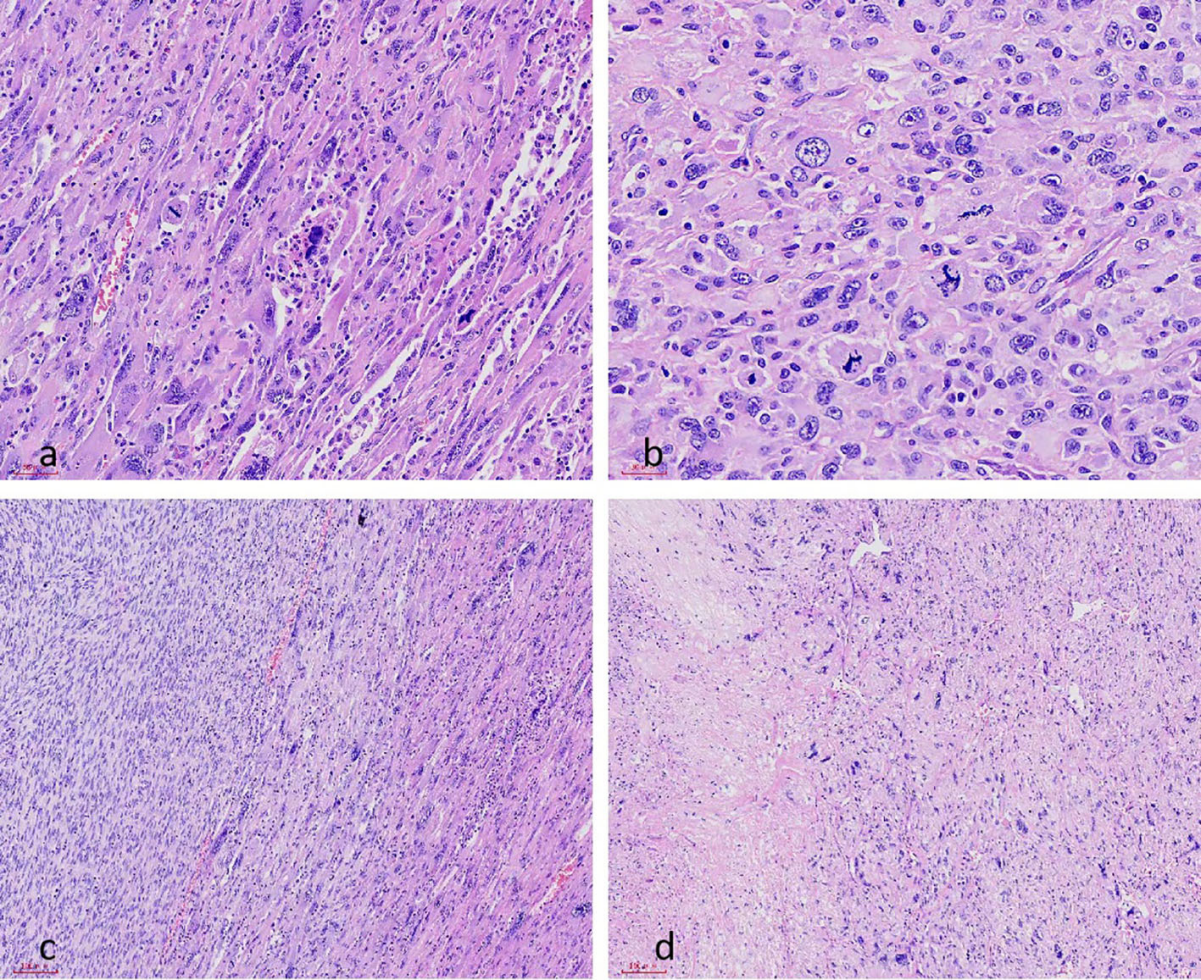
Histological finding of the dedifferentiated leiomyosarcoma in hematoxylin-eosin section.mitotic figures were shown under 20X **(a)**, mitotic figures were shown under 40X **(b)**, areas of spindle-shaped and osteoclastoid multinucleated cells **(c)**, Partially necrotic area of the tumor **(d)**.

**Figure 5:**
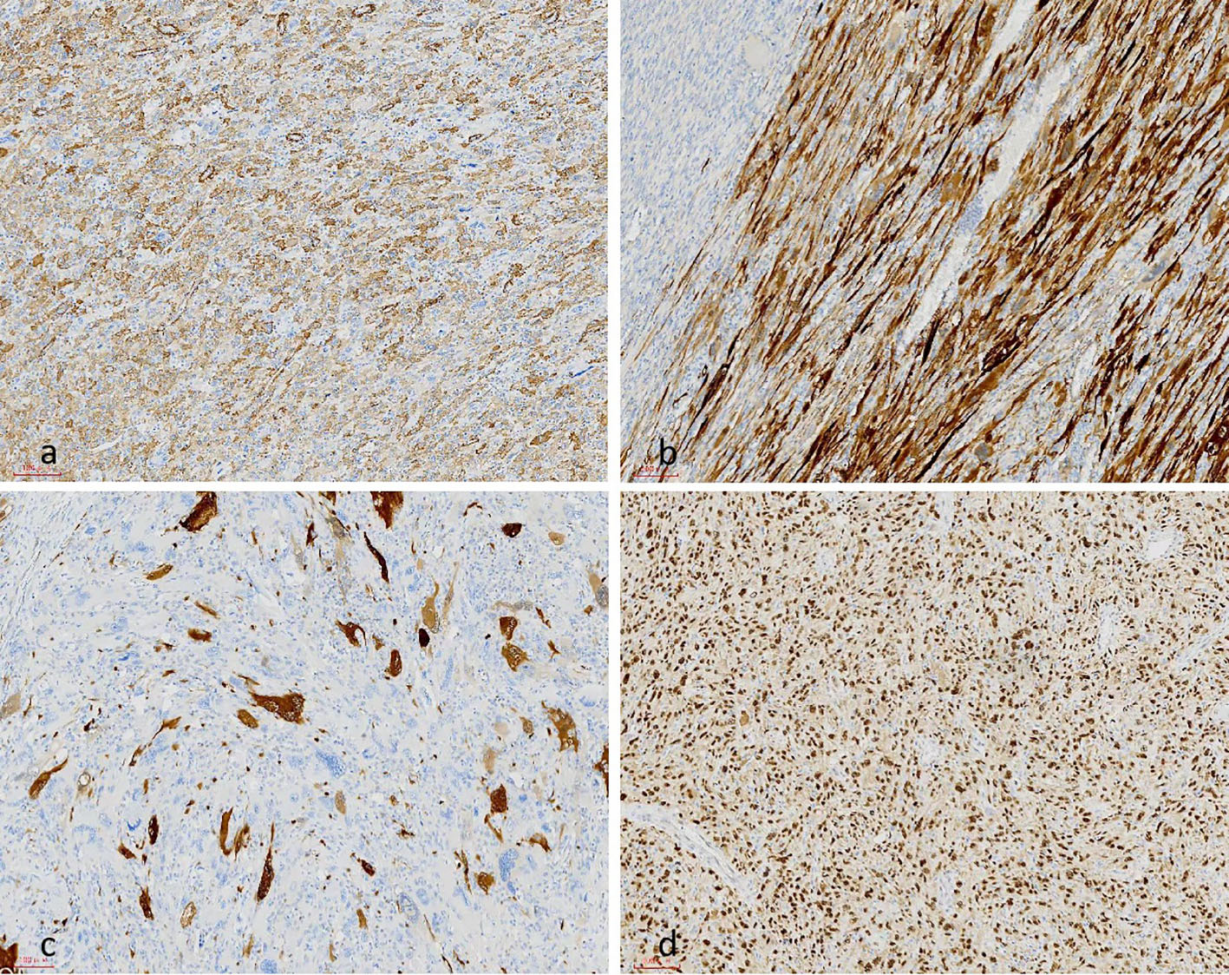
On immunohistochemical staining, the tumor cells were positive for SMA **(a)**, calponin **(b)** and P16 expression in a small number of cells **(c)**, and approximately 50% of cells were positive for P53 **(d)**.

Histopathological manifestations included cells with severe to marked anisocytosis accompanied by necrosis, >10/HPF nuclear division, and some tumor cells presenting pleomorphism and osteoclast-like multinucleated giant cell morphology, as well as nerve invasion (+). No tumor was observed at the surgical margin(**Figure 4**). On immunohistochemical staining, the tumor cells were positive for smooth muscle markers including smooth muscle actin (SMA), calponin and caldesmon, P16 expression in a small number of cells, and approximately 50% of cells were positive for P53. All additional stains, including S100, SOX-10, CK, EGFR, CD99, CD34, HMB, MyoD1, Bcl-2, STAT6, and Myogenin, were negative(**Figure 5**). This profile was consistent with a sciatic-confined leiomyosarcoma.

14 days after surgery, the patient’s red blood cell count, hemoglobin level, and albumin level remained close to normal levels. The swelling in the patient’s right lower limb was quickly relieved, and the patient was able to walk with the assistance of a knee brace. Postoperative motor function remained unchanged (BMRC grade 0 for ankle movement), but neuropathic pain improved (VAS 3/10). The patient received adjuvant radiotherapy for the affected limb based on the postoperative pathological diagnosis. The radiotherapy regimen consisted of a total dose of 60 Gy delivered in 30 fractions over 6 weeks, using intensity-modulated radiotherapy (IMRT) technique. The clinical target volume (CTV) included the tumor bed with a 3-cm craniocaudal margin and a 1.5-cm radial margin, with careful sparing of the surrounding skin and bone. The patient was followed up for six months after surgery. A follow-up MRI of the thigh and a CT scan of the chest at 6 months showed no evidence of local recurrence or distant metastasis.The neuropathic pain continued to improve (VAS 2/10). Although ankle motor function did not recover, the patient reported improved quality of life and independence in daily activities, as measured by a subjective improvement in the EQ-5D scale.

## Discussion

In this case report, we present a rare occurrence of primary sciatic leiomyosarcoma in a patient with neurofibromatosis type 1 (NF1), which to our knowledge, has seldomly been reported before in the literature. The association between NF1 and leiomyosarcoma is not well-established, with only a few case reports documenting this occurrence (8, 9). In this discussion, we will explore the possible mechanisms of the development of leiomyosarcoma in NF1 patients, as well as the diagnostic and management challenges presented by this rare occurrence.

NF1 is associated with a variety of malignant tumors, including malignant peripheral nerve sheath tumors (MPNSTs) (12, 13), brain tumors (14, 15), breast cancer (16), and hematologic malignancies (17). Additionally, individuals with NF1 may have an increased risk of developing rhabdomyosarcomas (18), pheochromocytomas (19), paragangliomas (19), gastrointestinal stromal tumors (20), and glomus tumors (21), which may occur more frequently than expected. Furthermore, some studies have reported an elevated susceptibility to leiomyosarcomas in individuals with NF1 (22).

While leiomyomas are uncommon in NF1, available literatures suggest that the association of NF1 and leiomyomas or leiomyosarcoma is not entirely coincidental. An intracranial leiomyosarcoma in a child with NF1 has also been previously described (23). Additionally, low-grade malignant peripheral nerve sheath tumor with smooth muscle differentiation has been reported in the literature (24). Several intestinal leiomyosarcomas have also been described in the literature, especially in the jejunum (25, 26)and duodenum (27).

The development of leiomyosarcoma in NF1 patients is thought to be related to the NF1 gene mutation, which affects the Ras-MAPK pathway (28). The Ras-MAPK pathway is a signaling pathway that regulates cell proliferation and differentiation, and its dysregulation has been linked to the development of cancer (29). The NF1 gene mutation leads to increased activity of the Ras-MAPK pathway, which in turn may contribute to the development of leiomyosarcoma (30).

Leiomyosarcoma typically does not arise from nerves. Rather, it arises from smooth muscle cells found in various organs such as the uterus, gastrointestinal tract, and blood vessels. Borvorn et al. reported a rare case of leiomyosarcoma arising from within the left sciatic nerve, which did not originate from tissue outside the nerve and invade it. The tumor was thought to have originated from a smooth muscle cell within the small vessel walls located outside the perineurium. The patient had NF1 and presented with weakness and numbness in the left foot and leg. The tumor invaded the epi-, pen-, and endoneuria of the left sciatic nerve trunk and was surgically resected (4).Another similar case was found in the literature, reported by Benyahya et al. Their patient had a leiomyosarcoma in the right buttock which infiltrated the right sciatic nerve, but the tumor was only partially excised and the patient’s health deteriorated with follow-up lost (5). Our case is similar to the case reported by Borvom, in which the tumor originated from the sciatic nerve and was speculated to arise from the vasa nervorum outside the perineurium. Intraoperative and histopathological evaluation confirmed that the tumor diffusely infiltrated the entire nerve trunk without selective fascicular involvement. The tumor grew by infiltrating and expanding along the nerve without invading the surrounding muscles, and had a complete capsule. During surgery, we attempted to separate the tumor from the nerve, but due to the absence of a clear boundary and significant bleeding, we had to remove both the tumor and the involved nerve tissue.

The histopathological distinction between leiomyosarcoma and malignant peripheral nerve sheath tumor (MPNST) with smooth muscle differentiation is crucial yet challenging. Our case demonstrated strong and diffuse positivity for smooth muscle markers (SMA, calponin, caldesmon) and negativity for S100 and SOX-10, which is characteristic of LMS and effectively rules out a conventional MPNST lineage (24). While rare cases of MPNST with heterologous smooth muscle differentiation exist, they typically retain focal expression of S100/SOX-10 in the Schwannian component (24). The immunophenotype in our case strongly supports an origin from the smooth muscle of the vasa nervorum, a hypothesis consistent with the case reported by Borvorn et al. (4). Furthermore, compared to the case reported by Benyahya et al. (5), where partial excision led to clinical deterioration, our patient achieved a favorable short-term outcome after R0 resection combined with adjuvant radiotherapy. This underscores the importance of complete surgical excision as the cornerstone of management for this rare entity. The overall prognosis for soft tissue leiomyosarcoma, especially in the extremity, is influenced by tumor size, grade, and margin status (3). Our patient’s large tumor size confers a higher risk of recurrence, necessitating close long-term surveillance.

To better contextualize our findings within the existing literature, we have summarized the previously reported cases of leiomyosarcoma arising in patients with NF1 in **Table 1**. As shown, primary sciatic nerve involvement, as seen in our patient, is exceptionally rare, with only two prior cases documented (4, 5). The table highlights the heterogeneity of tumor locations in NF1-associated LMS, including peripheral nerve, gastrointestinal tract, bone, and even intracranial sites. A comprehensive review by Afşar et al. (11) identified 15 additional cases from the literature, further supporting the association between NF1 and leiomyosarcoma. Our case adds to this limited body of literature by providing detailed documentation of surgical management with R0 resection, a standardized adjuvant radiotherapy regimen, and short-term follow-up outcomes. The favorable short-term outcome in our patient, compared to the deterioration seen in the case reported by Benyahya et al. (5), underscores the importance of complete surgical excision when feasible.

**Table 1:** Summary of previously reported cases of leiomyosarcoma associated with neurofibromatosis type 1.

Reference	Age/sex	Tumor location	Treatment	Outcome
Borvorn et al., 2003 (4)	51/M	Left sciatic nerve	Surgical resection	Decreased sensation intact after resection
Benyahya et al., 1997 (5)	22/F	Right buttock (infiltrating sciatic nerve)	Partial resection + radiation therapy	Tumor progression at 6 months, health deteriorated
Jhas et al., 2009 (23)	14/M	Intracranial (right temporal lobe)	Partial resection (hematoma evacuation, tumor not removed)	Hemodynamically unstable intraoperatively, tumor not fully resected
Ishizaki et al., 1992 (25)	54/F	Small intestine (jejunum)	Surgical resection (two separate occurrences, 13 years apart)	Second leiomyosarcoma arose 13 years after first resection
Miyoshi et al., 1996 (26)	49/M	Jejunum	Surgical resection	Tumor resected (mixed tumor with sarcomatous components)
Kawano et al., 1995 (27)	62/M	Duodenum (second portion)	Segmental resection (preserving pancreatic head)	Surgical margin free of tumor cells
Abualjubain et al., 2021 (8)	14/M	Bone (femur)	Biopsy confirmed, treatment not specified	Presented with 4-month history of pain and swelling
Afşar et al., 2013 (11)	Review article	Multiple sites (15 cases reviewed)	Various	Summarized 15 cases from literature
Present case	56/F	Right sciatic nerve	En bloc resection + adjuvant radiotherapy (60 Gy)	6 months follow-up, no recurrence or metastasis

*M, male; F, female.

The management of leiomyosarcoma in NF1 patients is challenging due to the lack of sufficient data supporting the optimal treatment approach. Surgery is the primary method for treating localized disease, but the role of chemotherapy and radiation therapy is not well-established. Some literature has reported more certain treatment outcomes when adjuvant radiation therapy is given after surgery (31). For localized soft tissue sarcomas, including LMS, current guidelines recommend wide surgical resection followed by adjuvant radiotherapy for high-risk cases to improve local control (31, 32).In this case, our patient underwent a partial sciatic nerve resection, followed by adjuvant radiation therapy. The patient survived for six months during follow-up, but the long-term survival and risk of recurrence remain unknown due to the short follow-up period. Furthermore, due to the rarity of this condition, further research is needed to determine the optimal treatment approach.

In conclusion, we report a rare occurrence of primary sciatic leiomyosarcoma in a patient with NF1, which highlights the importance of considering this diagnosis in NF1 patients with limb masses. Further research is needed to understand the underlying mechanisms of the development of leiomyosarcoma in NF1 patients, and to determine the optimal treatment approach for this rare occurrence.

## Data Availability

The original contributions presented in the study are included in the article/supplementary material. Further inquiries can be directed to the corresponding author.
